# Seven-Day Newspapers

**Published:** 1899-05-20

**Authors:** 


### The Hospital

A Journal of

The Medical Sciences and Hospital Administration.

Vol. XXVI.—No. 660.] Edited by Sir Henry Burdett, K.C.B., and Solomon C. Smith, M.D., M.R.C.P. [May 20, 1899.

### Seven-day Newspapers.

The very large and influential deputation on the subject of the so-called "seven-day" newspapers which was received by the Home Secretary last week no doubt represented a very considerable body of cultivated opinion. It is not every cause which could unite together persons so widely different, in many respects, as Mr. John Burns, M.P., and the Bishop of London, the Rev. Hugh Price Hughes and Cardinal Vaughan, Earl Percy, M.P., and a representative of the Amalgamated Society of Engineers. Nor need we attempt to conceal what is, perhaps, manifest, namely, that the opposition to the new form of enterprise, and the reasons advanced for discouraging it, were as various as the constitution of the deputation itself. Some of the speakers were influenced almost exclusively by the religious aspect of the question, others almost exclusively by the secular aspect; but it is not too much to say that there was general agreement in the opinion that the publication of Sunday editions by established daily papers hitherto confined to "working" mornings would, if not immediately, yet at no distant date, make appreciable inroads upon that system of a weekly holiday which, whether we regard it under a religious or a social aspect, is undoubtedly one of the most precious of the possessions of mankind. The great omnibus companies find it to their interest entirely to withdraw each of their horses from work, if not on one day in seven, at least on one day in nine; and the half-crazy doctrinaires of the French revolution, when they set up their new calendar, were not restrained by any fear of superstitious adherence to old usages or to the "*stile-esclave*" from determining that the *Décadi*, or tenth day of each month, *Brumaire*, *Pluviose*, *Floréal*, *Thermidor*, and the others, should always be a day of rest. It may be said, in fact, that a knowledge of the necessity of such days is as old as the experience of mankind, and that the institution of the Mosaic Sabbath was as much a consequence as a cause of this experience. Whether we regard the subject from a theological or from a purely social standpoint, it does not seem to be doubtful that a regularly recurring period of repose from labour is of the highest importance to the welfare of the race, and is an absolutely necessary condition of the fullest development of the powers of the individual. We cannot afford to surrender on this question any part of that national birthright under the influence of which our existing industries have acquired their present dimensions, and our capacities for work have attained their present standard. Considerations alike of public health and of public morality are totally opposed to what on one hand would be called the desecration of the Sabbath, and, on the other, its abandonment.

An endeavour has been made, by some of the advocates of the new departure in journalism, to show that no desecration of the Sabbath is involved in it, on the ground that the work would be done on Saturday, and that the papers now issued on Monday mornings are the results of Sabbath labour. This is perfectly true, in a large degree, as regards the supply of material to the compositors, and still more as regards the setting up of this material in type. But the journalist employed on a daily paper obtains his holiday on the Saturday of each week instead of on the Sunday; and suffers disadvantage, if at all, only by reason of the difference between Saturday and Sunday religious services. At first, no doubt, arrangements will be made by which the production of a Sunday edition of a daily paper will not be allowed to tax too heavily the time and thoughts of those who have been employed on the same columns during the week. The question is, how long such arrangements would endure in the face of competition. Many pressmen would be not only willing, but perhaps eager, to work continuously night after night for the sake of increased remuneration; and, prior to experience, would expect to relieve themselves from the consequences by a somewhat longer complete holiday at the slack time of the year. Every medical man knows that they would infallibly break down in the attempt, and that the annual holiday, if indeed it came at all, would not infrequently come too late to compensate for the strain to which the faculties had in the meantime been exposed. But, after all, the actual journalists and printers form but a portion of the vast army which is engaged in the diffusion of each day's intelligence. There are in England about 25,000 newsagents, employing about 100,000 boys as distributors, to every one of whom the increased issue of Sunday papers would mean a curtailment of their present scanty leisure, and a loss of the benefits which that leisure affords. The Home Secretary clearly conveyed to the deputation that they must not expect any impediments to the new enterprise from legislation, although both he and the Government generally were in sympathy with the representations made to him. The only course was to appeal to public opinion, in the hope that its response might be of such a nature as to show that the proposed innovation would be unprofitable. In this appeal, made in the interests of the existing weekly holiday, we feel it our duty to join with whatever force our representations may command. Seven-day newspapers would soon come to mean continuous labour; and continuous labour would be a national misfortune. We therefore hail with great satisfaction the announcement that Messrs. Harmsworth have already discontinued the issue of the *Sunday Daily Mail.*

ANNOTATIONS—*continued.*

supplied. These are matters the discussion of which too often leads to angry words and unseemly recrimination. It is sufficient to point to the difficulty and delay which, almost of necessity, must occur in obtaining medicine "out of hours" when the medical man has not the dispensing arrangements under his own control. Even in towns of considerable size it is often far more difficult to get the physic than the physician, and the smaller the place the more pronounced does this difficulty become. Nor can we entirely hide from ourselves the fact that in villages and little towns, where tittle tattle reigns supreme, serious trouble might arise were the local medical man to put himself entirely in the hands of the local chemist in regard to the dispensing of his medicines. In large towns and in consulting work the relations between the doctor, the patient, and chemist are of a curiously impersonal nature; but in small places all this is very different, and it would be easy to imagine cases in which the handing of a prescription to a chemist to dispense might come very near to a breach of professional confidence. So far as regards any supposed danger to the public involved in the continuance of the present system, or want of system, we fancy that the matter may safely be dismissed, as has been done by the General Medical Council and the Government. The active personal supervision of the master is likely to afford greater security than would attend the delegation of responsibility to a servant, however well qualified. In answer to Major Rasch, Sir J. Gorst said that the infrequency with which accidents were reported to the Privy Council tended to confirm the opinion of the General Medical Council that they were very rare, and the Privy Council had not, therefore, thought it necessary to ask the General Medical Council for any further information on the subject.

### Organisms of Disease.

It has been long customary to divide bacteria into two main classes, those which are capable of exciting disease in men and animals and those which do not possess this power. The tendency of recent bacteriological researches, however, is to show that no such clearly defined distinction can be made, for not only can many pathogenic bacteria be cultivated on various media, but under altered circumstances of nutrition and environment saprophytic and nonpathogenic bacilli seem capable of taking on true parasitic and pathogenic properties. Vincent has made an interesting series of experiments with two varieties of bacteria—the Bacillus megatherium commonly found in garden soil, and the potato bacillus (Bac. mesentericus vulgatus). Both these organisms have up to the present time been regarded as pure saprophytes, which when inoculated into animals give rise to no symptoms of disease. By successive cultures within collodion capsules placed in the peritoneal sac of guinea pigs it has now been found possible to completely alter the characters of these organisms. Their method of growth in all media becomes totally unlike what it was formerly. Previously aërobic they now grow equally well in vacuo, and if injected into animals excite a specific disease which is fatal in a few hours. The change from the saprophytic to the parasitic stage has been coincident with a complete alteration in the morphology and functions of the organism. These experiments have more than an academic interest, for they throw a light upon the very obscure question of the origin of diseases, and help to explain the return of certain epidemics and isolated outbreaks of disease. A germ inhabiting soil or water may through countless generations be innocuous until, under altered conditions, its characters become changed, and it acquires a virulence of such intensity as to excite disease processes in the human subject or in animals. As we have already pointed out, these facts will, if substantiated, do much to explain the mystery which surrounds the occurrence of some sporadic cases of typhus fever, and of localised and otherwise inexplicable outbreaks of enteric fever and diphtheria. They also accentuate the necessity for hygiene and cleanliness in life, for it is to be noted that these organisms, and we may also presume the organisms of all diseases, are foreign to the human economy, being introduced as extraneous matter from without.

### Little White Slaves.

So much attention has been paid of recent years to the study of the mental and physical condition of children of school age that most school inspectors and teachers, medical men, and visitors are able to detect the signs of nervous fatigue and exhaustion which occur in scholars on whom the work of the elementary schools presses too heavily. By careful selection and arrangement of the subjects taught, by diminishing the school hours, and by providing special classes for the instruction of the mentally deficient, much has been done to lessen the evils resulting from over-pressure. There still remain a large number of children, however, who, owing to insufficient food and physical exhaustion consequent upon long hours of labour, are incapable of receiving the full benefit of School Board training. Sir John Gorst, in his recent report to the House of Commons, stated that there are 145,000 children on the books as full-time scholars who are also engaged as labourers for wages or profit. Of these children 131 are under six, 1,120 between six and seven, 4,211 between seven and eight, 11,027 between eight and nine, and 22,131 between nine and ten. The hours of labour of the children under six years of age varied from 20 to 35 per week, and their earnings from 8d. to 3s. 6d. Amongst the older children the principal occupations were hawking, newspaper selling, message and errand work, laundry work, needlework, and tending babies. In some cases the hours of employment reached as high a figure as 70 or 100 per week, but the wages seldom exceeded five shillings. To some of the lighter forms of occupation, entailing only a few hours' labour in the week, there can be no serious objection; but it is obvious that employment necessitating close confinement to the house, late hours at night or very early rising, must even in the case of the older and more robust children be followed with grave detriment to the physical welfare of the children. Consequent upon the physical strain there is a corresponding lack of progress in their educational advancement, as is evidenced by the fact that 329 were classed as in no standard, 3,890 in Standard I., 11,626 in Standard II., 24,624 in Standard III., and 36,907 in Standard IV. To secure an efficient remedy for the condition revealed by the above facts will be a difficult problem for the Education Department to solve. On two points all persons probably will be agreed—namely, that a limit shall be placed upon the number of hours during which children attending school may be employed, and that a minimum age shall be fixed. It is a disgrace to our humanity that it should be possible for a school manager to write that "there are to-day in our national and Board schools thousands of little white slaves."

